# New MRI Criteria for Successful Vaginal Breech Delivery in Primiparae

**DOI:** 10.1371/journal.pone.0161028

**Published:** 2016-08-17

**Authors:** Janine Hoffmann, Katrin Thomassen, Patrick Stumpp, Matthias Grothoff, Christoph Engel, Thomas Kahn, Holger Stepan

**Affiliations:** 1 University of Leipzig, Department of Obstetrics, Liebigstrasse 20a, 04103 Leipzig, Germany; 2 University of Leipzig, Department of Radiology, Liebigstrasse 20, 04103 Leipzig, Germany; 3 University of Leipzig—Heart Center, Department of Radiology, Struempellstrasse 39, 04289 Leipzig, Germany; 4 University of Leipzig, Institute for Medical Informatics, Statistics and Epidemiology, Haertelstrasse 16–18, 04107 Leipzig, Germany; President, CMO, UNITED STATES

## Abstract

**Background:**

Even if lower vaginal delivery success rates and impaired neonatal short-term outcomes have been reported for primiparous women with breech presentation, vaginal breech delivery remains an option for carefully selected patients. Because Magnetic resonance imaging (MRI) pelvimetry can provide additional information on maternal pelvic morphology, we sought to identify new MRI parameters that predict successful vaginal breech delivery.

**Methods:**

In this retrospective unicentre study, 240 primiparous women with breech presentation at term underwent MRI pelvimetry. For all patients vaginal delivery was planned, according to German guidelines and if the conjugata vera (CV) was ≥12 cm. The patients with uneventful vaginal deliveries and the patients who underwent a secondary caesarean section were compared according to pelvimetric parameters and outcomes. Regression analyses were performed.

**Results:**

In the vaginal delivery group (n = 162, (67.5%)), the distance between the spinae ischiadicae (interspinous diameter, ISD) was significantly enlarged. The ISD significantly influenced the mode of delivery in the regression analyses. The CV did not significantly differ between the groups. The patients with successful vaginal deliveries were significantly younger than the patients who underwent caesarean section. In the receiver operating characteristic (ROC) analysis, the area under the curve (AUC) for ISD was 67.7% (p<0.001, 95% CI [0.303–0.642]) and was higher considering the mother’s age (AUC = 73.1%, p<0.001, 95% CI [0.662–0.800]). The neonatal short-term outcomes were comparable in both groups.

**Conclusion:**

The additional use of ISD may predict successful vaginal breech delivery and may be superior to the CV, which is more commonly used.

**Trial Registration:**

DRKS00009957

## Introduction

Breech presentation occurs in3-5% of all pregnancies and is one of the most common causes for elective caesarean section, today. Breech delivery requires special management; however, the preparation and preselection of delivery method remains controversial [1–3]. Vaginal delivery is always an option, but the results and interpretation of the Term Breech Trial brought about incisive changes [4]. Even though this trial had significant problems in its methodology, the analysis demonstrated the superiority of caesarean section over vaginal breech delivery according to immediate neonatal outcomes. After the publication of this trial, caesarean section rates increased dramatically [5,6]. The subsequent flood of publications and the withdrawal of the Term Breech Trial due to methodical flaws revived the controversy [6–11]. Maternal and neonatal long-term outcomes are comparable for vaginal and caesarean section deliveries when patients are carefully preselected and adequately treated [3,12,13]. However, higher primary neonatal morbidity and lower success rates are associated with vaginal breech delivery, especially in primiparous women [10,11,14]. Therefore, vaginal breech deliveries should be managed only in perinatal care centres with an adequate interdisciplinary infrastructure and experienced teams.

For preselection, maternal and foetal risk factors are considered, but detailed protocols for planned vaginal breech deliveries differ on the international and national level [15–17]. The additional value of magnetic resonance imaging (MRI) pelvimetry for preselection has not been sufficiently evaluated. Although excluded from national guidelines, MRI pelvimetry is performed in many perinatal care centres and can facilitate decision-making in challenging situations, particularly in primiparous women. MRI pelvimetry was implemented first by Stark et al. in 1985 [18]. Because of its high accuracy, MRI obviates the need for X-rays [19–21] and apart from ultrasound is the cross-sectional imaging modality of choice in pregnancy. To date, only one prospective study has investigated MRI pelvimetry for the management of breech delivery [22]. This study showed a reduction in the emergency caesarean section rate when patients with one or more pathologic pelvic measurements were excluded from vaginal breech delivery. However, the impact of single pelvimetric parameters on the outcomes of vaginal breech delivery has not been analysed. In addition, pelvimetric reference values obtained from older X-ray studies and MRI studies with mixed inhomogeneous patient groups have not been evaluated adequately in the setting of vaginal breech delivery [19,21–24]. However, pelvic dimensions are useful when planning breech deliveries [25–27].

In this study, we sought to determine the relationship between comprehensive contemporary MRI pelvimetry and labour success in a homogenous group of nulliparous women with breech presentation.

## Materials and Methods

This study was approved by the local ethics committee of the University Leipzig and was performed in accordance with the ethical standards of the Declaration of Helsinki and its later amendments. All patients gave their written informed consent for the scientific use of their anonymised data prior to inclusion in the study. Data were anonymized and de-identified prior to analysis.

### Patients and clinical management

We retrospectively analysed 240 primiparous women with singleton breech presentation at term who presented for vaginal delivery between January 2006 and August 2014. The inclusion criterion was the woman’s wish to deliver vaginally. The exclusion criteria were the patient’s request for a caesarean section, suspected foetal or foetomaternal disproportion, an estimated foetal weight <2500 g or >3800 g, severe foetal or maternal diseases, prematurity (<35^+0^ gestational weeks) or a conjugata vera (CV) <12 cm according to national German guidelines [15]. An additional exclusion criterion was a contraindication for MRI. Secondary caesarean section was indicated during delivery for pathologic cardiotocogram (CTG), umbilical cord prolapse, footling presentation and obstructed labour in the dilation or expulsion phase. All parameters were compared between the vaginal and caesarean delivery groups.

### Anamnestic and clinical parameters

Anamnestic and clinical parameters were obtained from the electronic medical records in the ViewPoint and SAP 710 documentation systems and were analysed according to the following outcomes: mother’s age at delivery, gestational age at sonographic and MRI examination, gestational age at delivery, maternal height, maternal weight, maternal body mass index (BMI) and maternal diseases.

### MR imaging and data analysis

MRI examinations were performed at 37.5±1.6 gestational weeks using a 1.5T MRI system (Symphonie, Siemens Healthcare, Erlangen, Germany) with patients in the supine position. The integrated body coil was used. A T2-weighted half-Fourier acquisition single-shot turbo spin-echo (HASTE) sequence was performed in the sagittal orientation, and a T1-weighted spin-echo (SE) sequence was performed in the axial orientation. T1- and T2-weighted sequences were combined for diagnostic purposes. The slice thickness was 5 mm in both sequences. Special patient preparation and contrast agents were not necessary.

**Fig 1:**
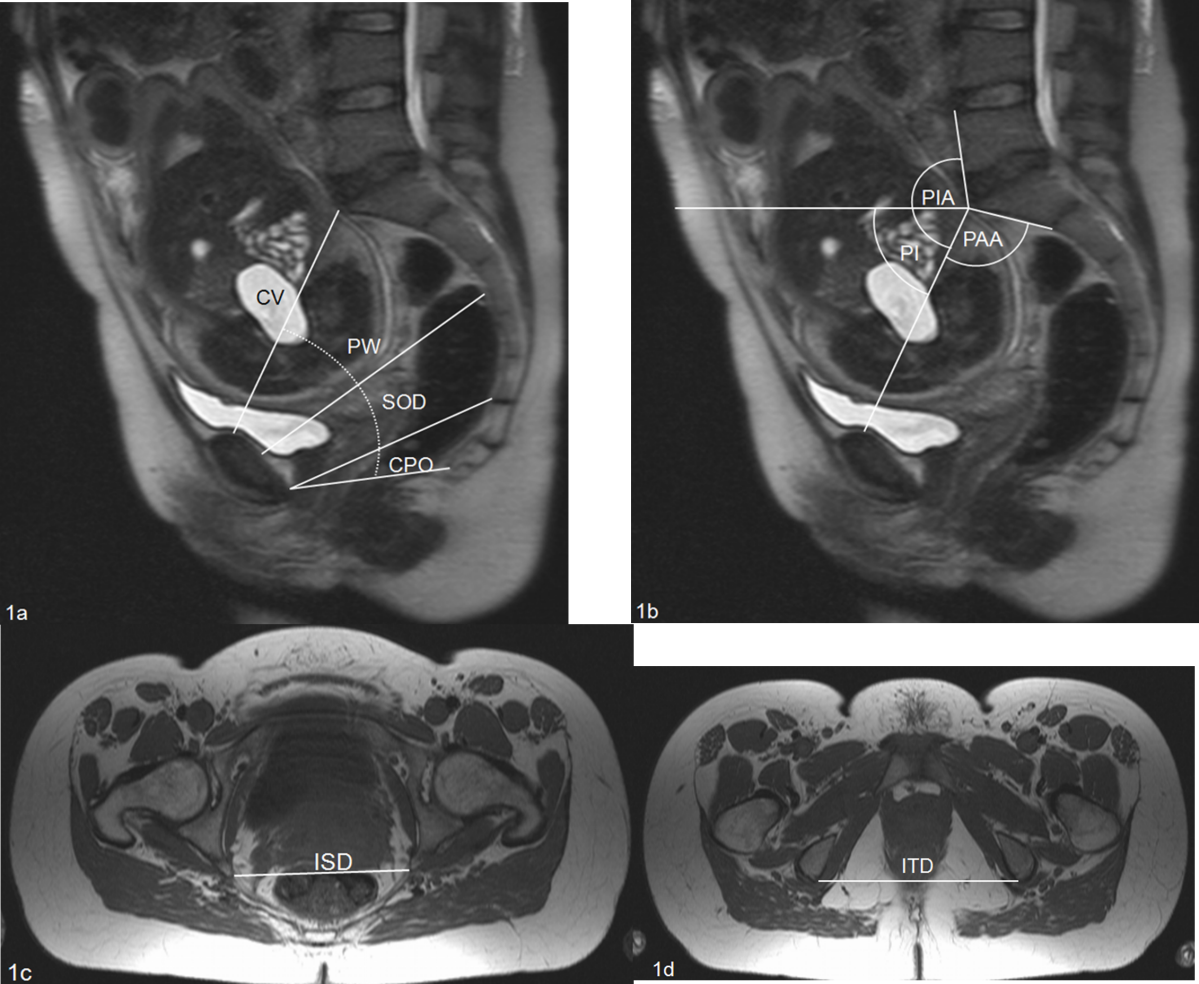
a: Sagittal distances. b: Sagittal angles.^18,24^. All distances were measured from cortex to cortex. CV = conjugata vera (cm): Sagittal distance between the promontory and the dorsal surface of the symphysis. PW = pelvic width (cm): Sagittal distance between the dorsal surface of the symphysis and the middle of the third sacral vertebra. SOD = sacral outlet diameter (cm): Sagittal distance between the lower margin of the symphysis and the sacral tip. CPO = coccygeal pelvic outlet (cm): Sagittal distance from the coccygeal tip to the lower margin of the symphysis. PAA = pelvic aperture angle (°): Angle between the CV and a line on the ventral surface of the first sacral vertebra. PIA = pelvic inlet angle (°): Angle between the CV and a line on the ventral surface of the caudal lumbar vertebra. PI = pelvic inclination (°): Angle between the CV and a horizontal line. Fig 1c+1d: Axial diameters.^18,24^. ISD = interspinous diameter (cm): Distance between the endpoints of the ischiadic spines. ITD = intertuberous diameter (cm): Distance between the posterior edges of both tubera ischii.

The pelvimetric dimensions were measured off-line on a separate workstation (Sectra PACS IDS5 11.4) by two blinded investigators with 8 (J.H.) and 3 (K.T.) years of experience in gynaecological MRI. Conventional diameters, including the conjugata vera (CV), the pelvic width (PW), the sagittal outlet diameter (SOD), the coccygeal pelvic outlet diameter (CPO), and the angles of the pelvic aperture (PAA), the pelvic inlet (PIA) and the pelvic inclination (PI) were measured in the sagittal orientation (Fig 1A and 1B). Pelvic outlet parameters, including the interspinous diameter (ISD) and the intertuberous diameter (ITD), were measured in the transverse orientation (Fig 1C and 1D). All MRI parameters were measured twice by two blinded observers: once to calculate interobserver variability and a second time to calculate intraobserver variability.

### Sonographic assessment

Sonography was performed using a GE ultrasound system (GE Healthcare, Voluson E8 Expert) with a 3.5 MHZ convex probe. The mean gestational age at the last sonographic examination was 39.8±1.3 weeks and did not differ between the vaginal and caesarean delivery groups (39.8±1.3 vs. 39.9±1.2, p = 0.568). Foetal weight was estimated using the Merz formula [28], which included the biparietal diameter (BPD) and the abdominal circumference (AC). The head circumference (HC) was calculated from the estimated BPD and the occipito-frontal diameter (OFD). Additionally, the femoral length was measured.

### Neonatal outcome parameters

In each delivery, the adaptation process of the neonate was monitored and attended by a paediatrician. Neonatal parameters were obtained, including head circumference, height, weight, pH of the umbilical cord, base-excess, 1-, 5- and 10-minute APGAR scores and neonatal intensive care unit (NICU) admission.

### Statistical analysis

The anamnestic, clinical, sonographic, MRI and outcome parameters were compared between the vaginal and caesarean delivery groups. Most parameters were normally distributed according to the Kolmogorov-Smirnov test, and the variances were homogeneous according to Levene’s test. Therefore, parametric tests were used, and the results were presented as the mean and standard deviation (SD). The categorical parameters were compared using the chi²-test or the Mann-Whitney test with the results expressed as the median (min/max; interquartile range, IQR). Bivariate correlations were tested using Pearson’s correlation. Univariate regression analyses were performed for all parameters that differed significantly between the vaginal and caesarean delivery groups. The parameters that significantly influenced the outcomes in the univariate regression analysis were included in the logistic multivariate regression analysis. For this analysis, the dataset was randomly split for model fitting (66%) and validation (34%). Odds ratios (ORs) were obtained from the logistic regression analysis. Receiver operating characteristic (ROC) curves and the area under the curves (AUCs) were used to describe the predictive performance of the models. P-values of 0.05 were considered to be statistically significant. The 95% confidence intervals (CIs) were obtained. Inter- and intraobserver variability was calculated for each MRI parameter. The reliability of the MRI measurements was tested by calculating the intraclass correlations (ICCs). Reliability was considered to be acceptable for ICCs ≥0.70, good for ICCs ≥0.80 and excellent for ICCs ≥0.90. IBM SPSS Statistics 20 was used for all the statistical analyses.

## Results

The characteristics of the patients are presented in Table 1, which compares the patients who underwent a vaginal delivery with the patients who underwent a caesarean section.

**Table 1:** The characteristics of the patients in the vaginal delivery and caesarean section groups.

	Vaginal delivery group N = 162	p	Caesarean section group N = 78
Age at delivery	28.8±3.9	**<0.001**	31.2±4.3
Gestational age at delivery	40.2±1.2	0.168	40.5±1.2
Maternal height	1.69±0.1	**0.007**	1.67±0.1
Maternal weight	62.6±9.7	0.853	62.4±10.5
Maternal body mass index	21.7±2.9	0.235	22.2±3.3
Maternal diseases	36/161 (24.4%)	0.770	19/79 (24.1%)

The mean maternal age at delivery was 29.6±4.2 years. The mean gestational age at delivery was 40.3±1.2 weeks.

Overall, 162/240 (67.5%) women had a successful vaginal delivery. Caesarean sections were medically indicated in 78/240 (32.5%) patients with pathologic CTG (n = 25/78, 32.1%), umbilical cord prolapse (n = 3/78, 3.8%), footling presentation (n = 5/78, 6.4%) or obstructed labour during the dilation (n = 5/78, 6.4%) or expulsion phase (n = 38/78, 50.0%). Labour was induced in 77/240 (32.1%) patients. The vaginal delivery rate was significantly higher in patients with spontaneous onset of labour (118/163, 72.4%) compared with patients who were induced (43/77, 55.8%, p = 0.011).

### MRI

The reproducibility of MRI was excellent for CV, PW and ISD and good for all other MRI parameters. The ICC coefficients for interobserver variability (the mean difference between two measurements from the two observers) were as follows: CV 0.97 (-0.09±0.24 cm), PW 0.97 (-0.12±0.24 cm), SOD 0.92 (-0.07±0.31 cm), CPO 0.83 (-0.04±0.6 cm), ISD 0.91 (0.02±0.38 cm), ITD 0.74 (0.12±0.8 cm), PIA 0.87 (-1.6±5.9°), PAA 0.89 (PAA 1.4±5.4°), and PI 0.95 (0.5±2.07°). The ICC coefficients for intraobserver variability (the mean difference between two measurements from one observer) were as follows: CV 0.96 (0.05±0.27 cm), PW 0.91 (0.12±0.26 cm), SOD 0.79 (-0.01±0.69 cm), CPO 0.76 (-0.07±0.75 cm), ISD 0.91 (-0.01±0.37 cm), ITD 0.73 (0.32±0.75 cm), PIA 0.89 (-0.27±5.4°), PAA 0.78 (-2.01±7.6°), and PI 0.86 (-2.5±2.6°).

The MRI pelvimetric parameters were compared between the vaginal and caesarean delivery groups. Only the ISD and the ITD differed significantly and were larger in the vaginal delivery group (Table 2). The mean differences between both groups were 0.52±0.11 cm for the ISD and 0.39±0.16 cm for the ITD. The CVs did not differ significantly between the groups.

**Table 2:** Comparison of the MRI parameters for the vaginal delivery and caesarean section groups.

MRI parameter	Vaginal delivery group N = 168	p	Caesarean section group N = 72
Conjugata vera (cm)	13.1±0.9	0.161	12.9±0.8
Pelvic width (cm)	13.7±1.3	0.063	13.4±0.9
Sagittal outlet diameter (cm)	11.5±1.0	0.290	11.3±1.0
Coccygeal pelvic outlet (cm)	8.6±1.0	0.511	8.5±0.9
Interspinous distance (cm)	11.1±0.8	**<0.001**	10.6±0.8
Intertubarous distance (cm)	13.6±1.2	**0.014**	13.2±1.1
Pelvic aperture angle (°)	87.7±11.0	0.365	86.3±10.6
Pelvic inlet angle (°)	144.2±14.6	0.604	145.2±11.7
Pelvic inclination (°)	66.1±9.1	0.118	64.4±6.4

**Fig 2:**
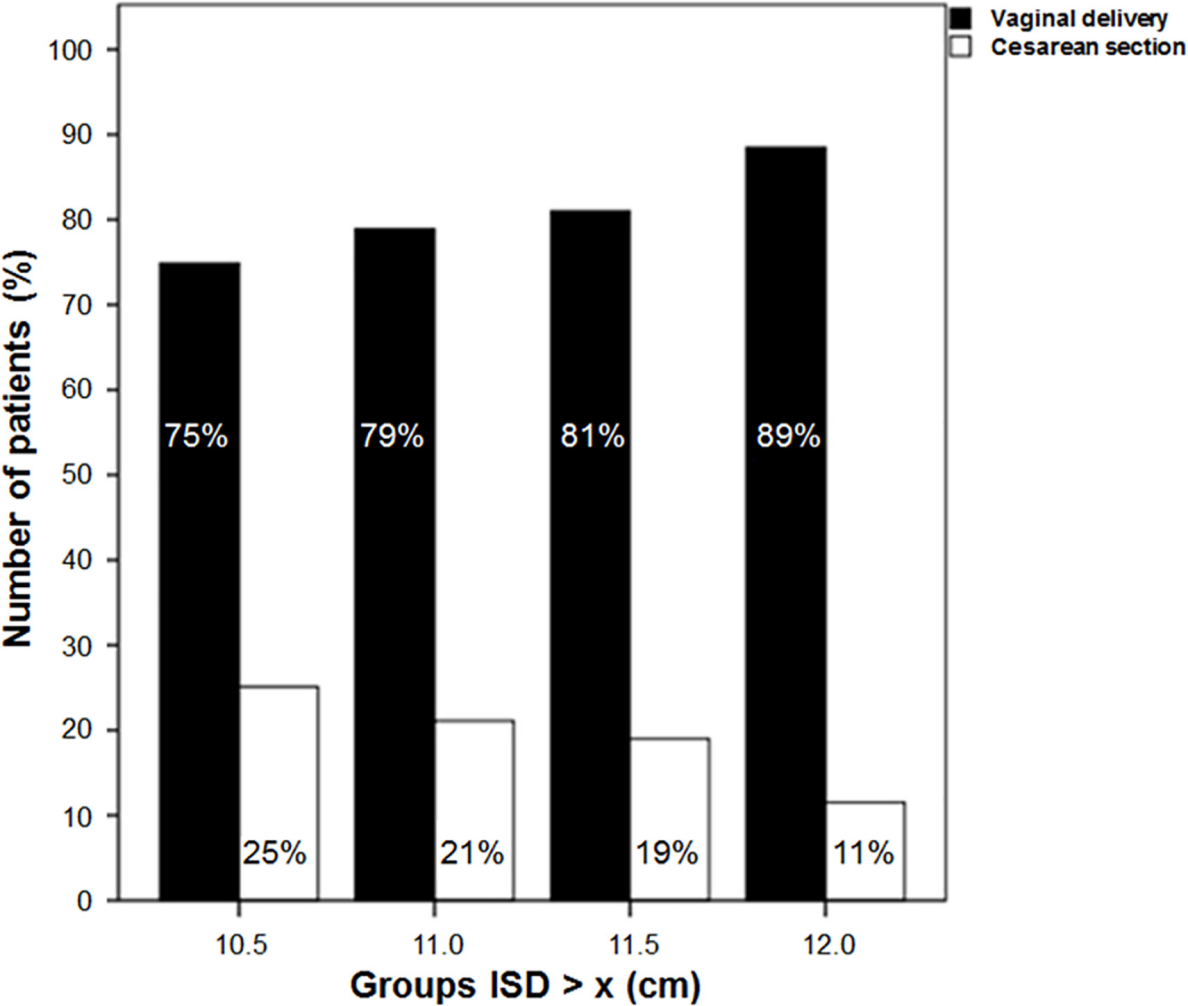
A comparison of the vaginal (black bars) and caesarean (white bars) breech deliveries according to the ISD. The study identified 175/240 (73%) patients with an ISD ≥10.5 cm, 109/240 (45%) patients with an ISD ≥11.0 cm, 63/240 (26%) patients with an ISD ≥11.5 cm and 26/240 (11%) patients with an ISD ≥12.0 cm.

In the univariate regression analyses, the ISD was the only MRI parameter associated with outcomes. Furthermore, the ISD had the greatest impact on outcomes in the multivariate regression analysis (Table 3). Fig 2 demonstrates the rate of successful vaginal breech deliveries for ISDs of 10.5, 11.0, 11.5 or 12.0 cm. If the lower reference value for the ISD of 11 cm was considered as an additional preselection criterion, 131/240 (55%) patients would have to be excluded from trial of vaginal breech delivery. 86 (79%) of the remaining 109 (45%) patients had a successful vaginal breech delivery and a caesarean section was indicated in only 23/109 (21%) of these patients. However, more patients (55/131, 42%) required a secondary caesarean section when the ISD was <11 cm. Overall, 58% of patients with an ISD <11 cm had a successful vaginal delivery (N = 76/131) and therefore would obtain an unnecessary primary caesarean section. These findings reflect a sensitivity of 71%, a specificity of 53%, a positive predictive value of 67% and a negative predictive value of 53%. Notably, CV was not associated with the mode of delivery in the univariate regression analyses (p = 0.161, OR 0.795, 95% CI [0.577–1.096]).

**Table 3:** Results of the univariate and multivariate regression analyses regarding successful vaginal breech deliveries.

	Univariate regression analysis	Multivariate regression analysis
Intertuberous diameter	0.062; 0.760 [0.570–1.014]	
Interspinous diameter	**< 0.001; 0.443 [0.305–0.644]**	**0.006; 0.468 [0.273–0.802]**
Estimated foetal weight (per 100 g)	**0.041; 1.093 [1.003–1.191]**	0.226; 1.081 [0.953–1.225]
Mode of birth	**0.012; 2.073 [1.177–3.651]**	0.083; 2.051 [0.910–4.622]
Mother’s age at delivery	**<0.001; 1.159 [1.079–1.245]**	**<0.001; 1.212 [1.092–1.345]**
Mother’s height	**0.008; 0.002 [0.000–0.197]**	0.569; 0.122 [0.000–169.980]

The bivariate correlations were significant for all the pelvimetric parameters but were strongest for ISD/ITD (R = 0.665; p<0.001), SOD/CPO (R = 0.661; p<0.001), PIA/PAA (R = 0.653; p<0.001) and PIA/PI (R = 0.514; p<0.001). The correlations between the ISD and the CV (R = 0.127, p = 0.049) and between the ISD and the mother’s height (R = 0.413, p<0.001) were significant but weak.

### Sonography

The sonographic parameters for the vaginal birth group and the caesarean section group are compared in Table 4.

**Table 4:** Comparison of the sonographic parameters for the vaginal delivery and caesarean section groups.

Sonographic parameter	Vaginal delivery group	p	Caesarean section group
Biparietal diameter (mm)	95±4	0.110	96±4
Occipito-frontal diameter (mm)	120±6	0.441	121±7
Head circumference (mm)	339±14	0.546	340.5±18
Abdominal circumference (mm)	315±18	0.088	319.5±17
Femoral length (cm)	73±3	0.119	74±3
Estimated foetal weight (g)	3165±3399	**0.040**	3262±320

### Neonatal outcomes

Neonatal outcomes were appropriate in both the vaginal and caesarean delivery groups (Table 5). No adverse foetal outcomes occurred.

**Table 5:** Comparison of the neonatal outcome parameters between the vaginal and caesarean section groups.

	Vaginal delivery group	p	Caesarean section group
Male/ Female	56/105		39/40
Head circumference (mm)	348.4±13.7	**<0.001**	355.5±14.8
Birth length (cm)	48.8±2.1	**0.003**	49.7±2.5
Birth weight (mm)	3231.1±386.8	**0.005**	3387.9±439.8
pH	7.18±0.1	**<0.001**	7.24±0.1
Base-excess	-6.93±3.8	**0.001**	-4.9±4.8
APGAR (1 minute)	8 [1/10; 2]	0.065	8 [1/9; 1]
APGAR (5 minutes)	9 [2/10; 2]	0.307	9 [6/10; 2]
APGAR (10 minutes)	10 [6/10; 1]	0.489	10[7/10; 1]

### Multivariate regression analyses

**Fig 3:**
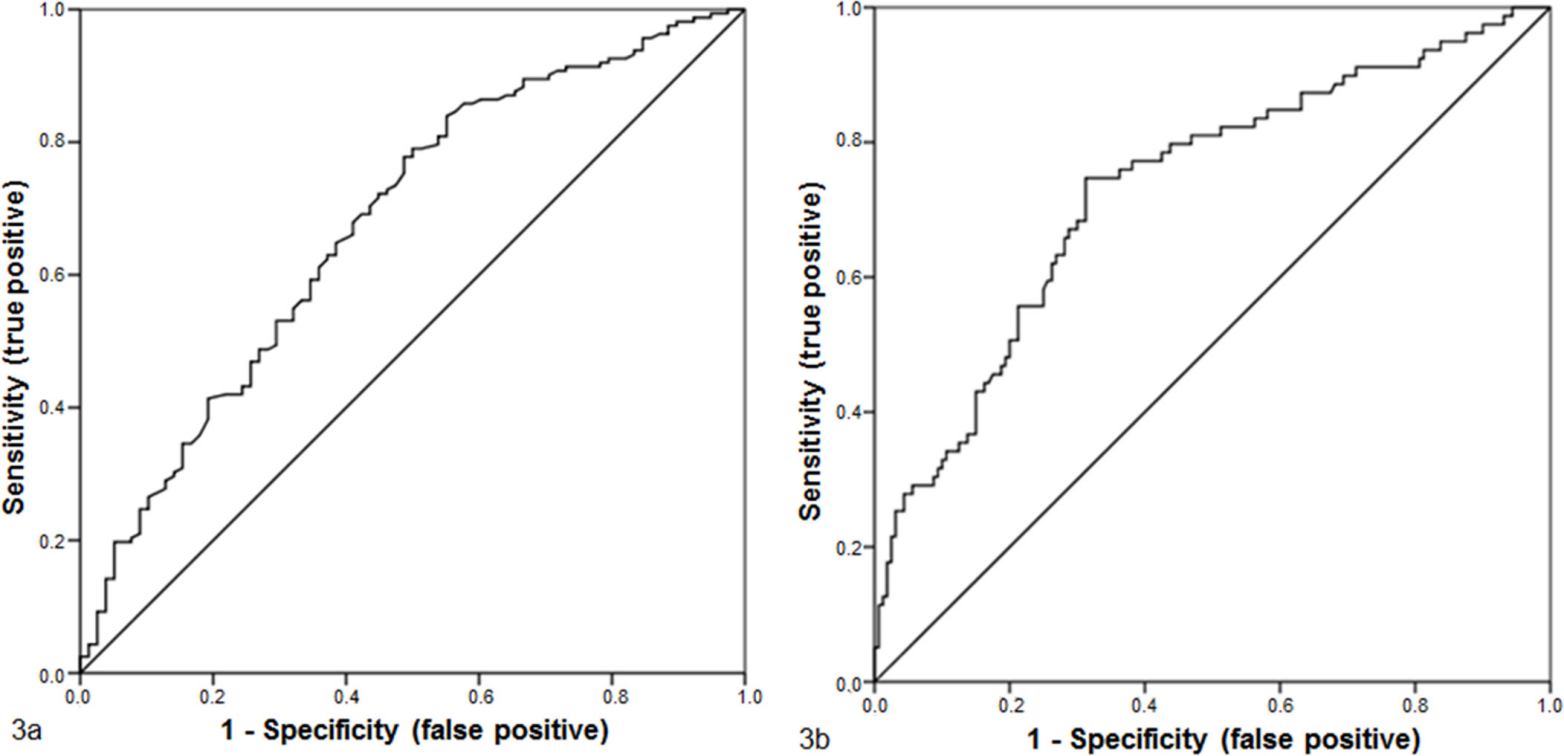
Figs 3a and 3b: The ROC analysis. The results of the ROC analysis show the significant effect of the ISD on vaginal breech delivery success. The AUC was 67.7%, and the regression equation was 8,164–0.818*ISD (Fig 3a). The AUC was 73.1% when maternal age was included in the ROC analysis. The regression equation was 2.949–0.859*ISD+0.193*mother’s age at delivery (Fig 3b).

In the multivariate regression analyses, only the ISD and the mother’s age were significantly associated with birth outcomes. The effect of the ISD on vaginal birth success was highly significant, with an AUC of 67.7% (p<0.001, 95% CI [0.604–0.750]). Regarding the maternal age at delivery, the AUC was 73.1% (p<0.001, CI 95% [0.662–0.800]) (Table 3, Fig 3A and 3B).

## Discussion

To the best of our knowledge, this study is the largest to analyse the relation of MRI-derived pelvic dimensions on birth outcomes in nulliparous women with a breech presentation at term. Our results show that the ISD may play a key role in the success of vaginal breech labour.

Historically, the CV is considered the most important pelvimetric preselection MRI parameter. Foetomaternal proportion is assessed using the CV and estimated foetal weight. A significant correlation between the CV, foetal weight and labour success was found in one retrospective X-ray study in 1975 [26]. Our results did not suggest that the CV and foetal weight could predict birth outcomes, which indicates that the role of the CV may be overestimated. In contrast, our data indicate that the stage of the lower midpelvic level is a more critical obstacle to overcome in breech presentation. The ISD reflects the space at this level. Recent publications support this hypothesis. Studies performed using open MRI systems gave new insights into pelvic biomechanics and delivery mechanisms. In non-pregnant women, CV, ISD and ITD dimensions change according to body position. In the kneeling position, the ISD and the ITD enlarge but, surprisingly, the CV shortens in contrast to the supine position [29,30]. This finding may explain the widespread use of the kneeling position during the pushing phase based on practical experience. Therefore, our data suggest that the ISD should be considered a useful preselection parameter when planning vaginal breech deliveries.

In the literature, ISD reference values range between 10.3 [23] and 11.6 cm [19]. However, these diameters were obtained from inhomogeneous study cohorts. Our study cannot provide concrete reference values, but a vaginal breech delivery success rate of 79% was achieved in patients with an ISD >11 cm. Compared with a success rate of only 67% when using conventional cut-offs, this increase is clinically relevant. With a positive predictive value of 67%, the ISD can be considered clinically valuable for further consultation and investigation, with the understanding that the birthing process is multifactorial. It has to be mentioned that 58% of patients with an ISD < 11 cm would obtain an unnecessary primary caesarean section when using ISD for prenatal preselection. For this reason we actually do not argue that the ISD is an exclusive selection parameter. In our opinion, the high positive predictive value of the ISD and the high success rates in patients with ISD ≥ 11cm rather justify the use of the ISD as additional orientating parameter for decision making. Compared with previously published studies, the vaginal delivery success rate in the present study was higher [6,10,31–33]. Remarkably, in these previous studies, the women were not consistently nulliparous and were not selected using pelvimetry. Thus, the higher success rate in our cohort may be explained by our preselection criteria using the CV. Because a weak correlation was found between the CV and the ISD, several patients with obstructed pelvic outlets may have been excluded. Nevertheless, nulliparous women with breech presentation are known to have significantly lower vaginal delivery success rates than multiparous women. Therefore, the high success rates in this study indicate the usefulness of MRI pelvimetric parameters as selection criteria for vaginal breech delivery.

The impact of the CV as a preselection parameter could not be adequately investigated in this study because the CV was used for patient selection. In the literature, CV reference values range between 11.3 [23] and 12.2 cm [19,21,23,24,26,34]. In our conventional selection protocol, the lower reference value for the CV is 12 cm.

Our study suggests that the ISD is highly correlated with successful deliveries in patients with breech presentation. Our results challenge the use of the CV as an appropriate selection criterion and suggest that historically established parameters, such as the CV, need to be revised. The midpelvic level and the flexibility of the female pelvis during birth should be considered selection criteria in a modern preselection protocol.

As an additional transverse MRI parameter of the pelvic outlet, the ITD is not considered a useful selection criterion due to the poor reproducibility and technical difficulty associated with measuring this parameter [21].

The high precision of MRI pelvimetry has been previously demonstrated, independent of the examiner’s experience or the patients’ constitution [18,23], and our results support these findings. The excellent precision of ISD measurements suggests that this parameter is an important selection criterion. Because of its safety, MRI is the optimal approach for pelvimetry during pregnancy. In previous studies, MRI measurements were demonstrated to be stable during pregnancy, delivery and after pregnancy [35] and were slightly lower in non-pregnant women [29]. We recommend the integration of MRI-derived pelvic dimensions into birth planning between the 35^th^ and 38^th^ gestational weeks in women with breech presentation.

The main limitations of this study are its retrospective design and the preselection of patients. Therefore, the impact of the sonographically estimated foetal weight and the CV on outcomes could not be adequately investigated. Because of missing data, indicator parameters for the disproportion between maternal and foetal pelvic dimensions, such as the foetal pelvic index [36], could not be included in our data analysis.

### Conclusion

Our study suggests that new MRI criteria are more closely associated with successful vaginal breech deliveries than pelvic measurements, such as the CV. The findings from this study indicate that the midpelvic level and the flexibility of the female pelvis during birth should be considered in a modern preselection protocol. The midpelvic level (ISD) reflects the pelvic space more accurately than a parameter of the pelvic entrance (CV). When larger than 11 cm, the ISD is a highly predictive factor for a successful vaginal breech delivery. This finding needs to be evaluated in a prospective study with a greater number of patients. The relevance of common metric parameters, such as the CV, should be reconsidered.
